# Efficacy of Seed Oils From *Azadirachta indica* and *Schinus molle* and Their Combination Against *Anopheles gambiae* s.l.

**DOI:** 10.1155/jotm/4460220

**Published:** 2025-09-15

**Authors:** Getnet Atenafu, Netuhsew Atnaf

**Affiliations:** Department of Biology, College of Natural and Computational Sciences, Debre Markos University, Debre Markos, Ethiopia

**Keywords:** *Anopheles gambiae s*.*l*., essential oil, extraction, larvicidal, mortality

## Abstract

Due to the increasing resistance to synthetic insecticides and the proliferation of resistant strains of malaria vectors, plant-derived essential oils (EOs) are gaining prominence as an alternative for controlling *Anopheles gambiae s*.*l*. larvae. This study evaluated the larvicidal efficacy of EOs from *Azadirachta indica* and *Schinus molle*, both individually and in combination, against early 4^th^-instar *Anopheles gambiae s*.*l*. larvae under laboratory and semifield conditions. The EOs were extracted using ethanol in a Soxhlet apparatus. Twenty-five treatments were arranged in a completely randomized design for experimentation. Temephos (Abate) and distilled water served as positive and negative controls, respectively. Each treatment contained 20 larvae, which were acclimatized for 2 h prior to exposure to EOs. Treatment cups were covered with muslin cloth to prevent debris contamination. Larval mortality was assessed at 24, 48, and 72 h postexposure at concentrations of 25, 50, and 75 ppm. In the laboratory, *A. indica* oil exhibited the highest larvicidal activity among plant treatments, with 71.66% mortality at 75 ppm after 72 h, while *S. molle* and the combined treatment produced moderate effects. LC_50_ and LC_90_ values confirmed *A. indica*'s superior potency, with significantly lower lethal concentrations across all exposure times (*p* < 0.05). In simulated field trials, *A. indica* oil also demonstrated time- and dose-dependent larvicidal activity, achieving 68.33% mortality at 75 ppm after 72 h. Synthetic larvicide Temephos (Abate) consistently caused 100% mortality within 24 h at all concentrations in both settings. These findings suggest that *A. indica* EO, particularly at higher concentrations and longer exposures, may serve as an effective botanical alternative for larval control in integrated vector management programs.

## 1. Introduction

Mosquitoes (*Diptera: Culicidae*) are primary vectors of critical vector-borne diseases such as malaria, lymphatic filariasis, Japanese encephalitis, dengue, yellow fever, and other forms of encephalitis [1]. Malaria, in particular, is transmitted to humans through the bite of female *Anopheles* mosquitoes carrying *Plasmodium* protozoa [2, 3]. In 2021, an estimated 247 million individuals were affected with malaria, resulting in 620,000 deaths globally, with Sub-Saharan Africa accounting for 95% of these cases [2].

Of the 156 *Plasmodium* species identified, five infect humans: *P*. *falciparum*, *P*. *vivax*, *P*. *ovale*, *P*. *malariae*, and *P. knowlesi* [4]. In Ethiopia, *P. falciparum* and *P. vivax* are the most epidemiologically significant, contributing 60% and 40% of malaria cases, respectively [5]. Malaria is one of Ethiopia's top public health concerns, causing approximately 1.2 million outpatient visits annually and negatively impacting socioeconomic activities [6, 7].

Among the 537 identified species of *Anopheles*, only 41 are known to transmit malaria under natural conditions [8, 9]. In Africa, 20 species serve as malaria vectors, with *Anopheles gambiae*, *An*. *arabiensis*, *An*. *coluzzii*, and *An. funestus* being the most significant vectors on the continent [10]. Ethiopia's primary malaria interventions include long-lasting insecticidal nets (LLINs), indoor residual spraying (IRS), larval source management, and effective case management using antimalarial drugs [11]. Despite these efforts, the rise in insecticide resistance among major malaria vectors jeopardizes vector control and elimination strategies [12, 13]. Therefore, there is an urgent need to explore alternative, eco-friendly insecticides.

Plant-derived insecticides, including essential oils (EOs), offer a biodegradable and nontoxic alternative that poses minimal risk to nontarget organisms. The EOs extracted from aromatic plants have demonstrated potential insecticidal activity against various vector species [14–16]. Several studies in Ethiopia have reported the larvicidal and adulticidal efficacy of plant extracts against *Anopheles arabiensis* [6, 17, 18], while *Schinus molle* extracts have shown activity against *Culex quinquefasciatus* larvae [19]. However, the larvicidal potential of EOs derived from *Schinus molle* and *Azadirachta indica* seeds against *Anopheles gambiae* remains unexplored. Therefore, the present study evaluates the larvicidal efficacy of EOs extracted from *S. molle* and *A. indica* seeds, both individually and in combination, against field-collected 4^th^-instar larvae of *Anopheles gambiae* under laboratory and semifield conditions.

## 2. Materials and Methods

### 2.1. Description of the Larvae Collection Site

**Figure 1:**
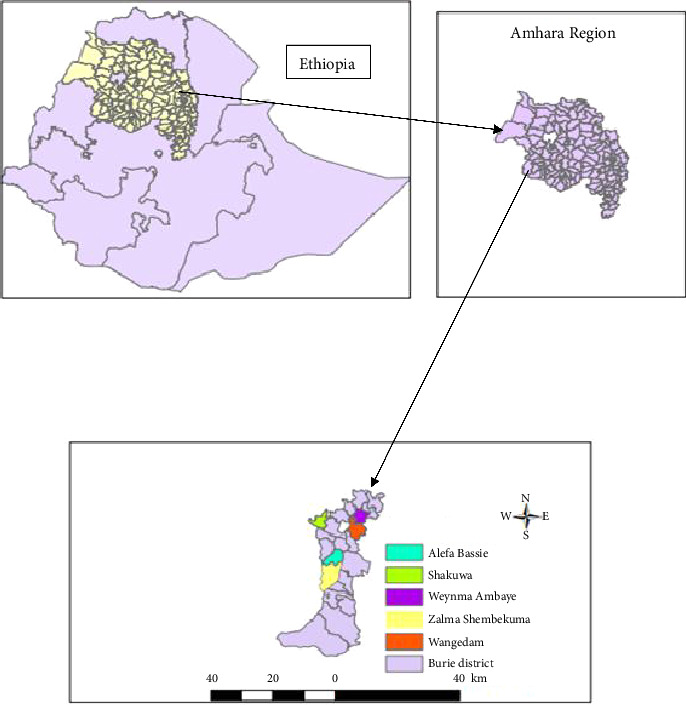
Study sample collection sites from Bure Zuria district, West Gojjam Zone, Amhara Region, Ethiopia.

Larvae of *Anopheles gambiae s*.*l*. were collected from Bure Zuria district, located in West Gojjam administrative zone of the Amhara Region, Ethiopia (Figure 1). The district is located 400 km north of Addis Ababa and 148 km southwest of Bahir Dar, the regional capital. Bure Zuria district ranges in altitude from 700 to 2350 m above sea level, with temperatures ranging from 9.9°C to 29.2°C and an annual rainfall of 1350–2500 mm. The rainy season typically occurs between July and September [20]. The district's economy is predominantly agricultural, with 85% of the population engaged in farming crops such as maize, pepper, potatoes, wheat, and millet. Samples were collected from five key breeding sites in the district: Alefa Bassie, Shakuwa, Weynma Ambaye, Zalma Shembekuma, and Wangedam. Semifield experiments were conducted at Zalma Shembekuma.

### 2.2. *Anopheles* Larvae Collection

Before immature collection, a total of 35 breeding sites in the Bure Zuria and Bure town districts were visited to conduct a larval survey. *Anopheles* mosquito larvae were collected over 10 days from major breeding sites. Collections were performed by the same individuals during the morning (9:00–11:00 a.m.) and afternoon (3:00–6:00 p.m.) using a World Health Organization (WHO) standard dipper (350 mL capacity, BioQuip Products, Inc., California, USA). When larvae were presented, the dipper was submerged gently at 45° about an inch below the surface of the water quickly with the sun in one's face. *Anopheles* larvae were carefully inspected for their presence. The larvae were then transferred into 10-liter plastic jars covered with muslin cloth for air ventilation, supplied with fish food (Tetra) until transportation to the Mosquito Insectary of Debre Markos University.

The collected samples were emptied into a white enamel tray. The larvae were then maintained under laboratory conditions at 27°C and 60%–70% relative humidity. *Anopheles* larvae were sorted from Culicine larvae and counted, recorded, and identified using Gillies and Coetzee's key [21]. The larval instar stages were determined based on size and morphological features as described by Walker and Lynch [22] at the Debre Markos University Entomology Laboratory.

### 2.3. Collection of Test Plant Components and Seed Powder Preparation

Seeds from selected plants were collected on 5 January 2020, from the Amhara Forest Enterprise Regional Seed Center in Bahir Dar, Ethiopia. The research methods were carried out according to the relevant guidelines and regulations of Debre Markos University research guidelines. The plants were already identified by the AFERSC and confirmed by Dr. Getaneh Belachew (Botanist) at Debre Markos University. Plant parts were transported separately in plastic bags to the laboratory for extraction. The plant voucher specimen was deposited at the herbarium with ID 0021 and complies with the university guidelines without the need for further affirmation. The experimental protocols for this research were approved by the Debre Markos University Research and Technology Transfer Directorate. The seeds were scrubbed and rinsed thoroughly to remove dirt and impurities, and then dried in an oven at 50°C to achieve a consistent moisture content. The dried seeds were finely ground using a mortar and pestle. The resulting powder was stored in airtight glass jars in a refrigerator until it was ready for oil extraction.

#### 2.3.1. Seed Oil Extraction

Seed oils from *A. indica* and *S. molle* seed powders were extracted using the Soxhlet extraction method with ethanol as the solvent in the chemistry laboratory at Debre Markos University. Equipment used included analytical balances, filter paper, a heater mantle, a Soxhlet chamber, conical flasks, and volumetric flasks. For extraction, 100 g of *A. indica* and *S. molle* seed powder were weighed and placed in a thimble with 500 mL of ethanol. The distillation process was conducted for 3 hours. After extraction, the solution was placed in a water bath to evaporate the solvent, leaving the EO.

### 2.4. Study Design

The study aimed to evaluate the larvicidal properties of seed oils from *A. indica* and *S. molle* seeds, both individually and in combination. The experiment followed a completely randomized design (CRD) with five treatments. Seed oils were applied at three concentrations: 25 ppm, 50 ppm, and 75 ppm (0.0025%, 0.005%, and 0.0075%), which were selected based on preliminary range–finding tests and literature reports, to ensure appropriate mortality levels for dose–response analysis. Each treatment had three replications, including a negative control (distilled water) and a standard control (Temephos/Abate) with the chemical name O,O′-(thiodi-4,1-phenylene) bis(O,O-dimethyl phosphorothioate). After larval identification, twenty 4th-nstar *An. gambiae* s.l. larvae were acclimatized in the lab for 2 hours before treatment application. The experiment was conducted under a photoperiod of 12:12 h (light: dark) at 27 ± 3°C and 70% relative humidity.

### 2.5. Preparation of Stock Solutions and Dilutions

To prepare a 1% stock solution, 1 mL of EO was mixed with 1 mL of ethanol in a graduated cylinder, stirred well to ensure complete mixing for 30 s, and diluted with distilled water to a total volume of 100 mL. The solution was labeled and stored in a sterile screw-cap glass vial with aluminum foil. Serial dilutions were prepared in the laboratory using micropipettes to achieve concentrations of 25, 50, and 75 ppm. These were made by diluting 1.125, 0.75, and 0.375 mL of stock solutions into 148.875 mL, 149.25 mL, and 149.625 mL of distilled water, respectively. Dilution was performed using the formula: C_1_V_1_ = C_2_V_2_, where C1 is the concentration of the stock solution, V1 is the volume of the stock solution required, C2 is the desired concentration of the dilution, and V2 is the final volume of the dilution. Positive control solutions consisted of 149 mL of distilled water mixed with 1 mL of ethanol, while 1% Temephos stock solution was diluted similarly for comparison.

### 2.6. Larvicidal Bioassay in the Laboratory

Larvicidal toxicity tests followed the WHO guidelines [23] for mosquito larvicides. White 250-mL cleaned test cups with 100 mL of distilled water were prepared, and 20 early 4th-instar *An. gambiae* s.l. larvae were placed in each cup after a 2-h acclimatization of the larvae. Each cup was exposed to the test solutions (25, 50, and 75 ppm). An untreated check was prepared with 150 mL distilled water. The cups were covered with muslin cloth and maintained at 25 ± 2°C, 80 ± 10% relative humidity, and a 12:12-h light: dark photoperiod. Mortality was assessed at 24, 48, and 72 h. Larvae were considered dead if they showed no movement when prodded twice with a needle. The dead larvae were counted in each treatment and were promptly removed to prevent decomposition. Each treatment was repeated three times, and due to larval scarcity, the bioassays were conducted over a one-week period using freshly prepared solutions for each test.

### 2.7. Larvicidal Bioassay Under Semifield Conditions

The EO from *A. indica*, which exhibited strong larvicidal activity in the laboratory, was further evaluated under semifield conditions from September 21 to October 19, 2021, in the Bure Zuria district. White 250-mL test cups were partially buried in marshy hay and leaves in a shaded, mosquito-prone area and cups were filled with 100 mL of distilled water. The stock solution and dilution of EO were the same as the laboratory experiment. Temperature and humidity were measured using a thermohygrometer. Twenty field-collected early 4th-instar larvae were added to each cup, treated with EO, and covered with muslin cloth. Control groups included Temephos and distilled water.

Similar to the laboratory procedure, larvae were considered dead if they showed no movement when prodded twice with a needle. The dead larvae were counted in each treatment and were promptly removed to prevent decomposition. Each treatment was repeated three times. Mortality was recorded at 24, 48, and 72 h.

### 2.8. Data Analysis

Mortality data from all replicates were pooled and analyzed using ANOVA in SPSS Version 25. Mean values were compared using Tukey's honestly significant difference (HSD) test at a significance level of *p* < 0.05. Probit analysis was performed to calculate LC_50_ and LC_90_ values. No control larval mortality was observed after 24 h, so correction based on Abbott's formula was unnecessary.

## 3. Results

### 3.1. Larvicidal Effects of Seed Oils Against *Anopheles gambiae s.l* Under Laboratory Conditions

The larvicidal effects of different concentrations of seed oils were tested against *Anopheles gambiae* s.l. larvae. The observed mortality rates (%) after 24 h are summarized in Table 1. The highest mortality rate of 71.65% was observed with *A. indica* seed EO at a concentration of 75 ppm after 72 h of exposure. This was followed by a mortality rate of 55% in the combined treatment of *A. indica* and *S. molle* oils under the same conditions. Statistical analysis revealed a significant difference (*p* < 0.05) in mortality between the treatments and control groups across all tested concentrations and exposure durations.

### 3.2. Determination of LC_50_ and LC_90_ Values of Seed Oils Against *An. gambiae* s.l. Under Laboratory Conditions

The dose–response relationship and the estimated LC_50_ and LC_90_ values with 95% confidence limits for the seed oils and their combinations, with exposure times of 24, 48, and 72 h, against early 4th-instar field-collected *An*. *gambiae s*.*l*. larvae, are presented in Table 2. The time-dependent changes in LC_50_ and LC_90_ values within each treatment reflect the progressive efficacy of the formulations over time. Comparisons between treatments at each time point revealed that *A. indica* consistently exhibited lower LC_50_ and LC_90_ values than *S. molle* and the combined treatment, indicating greater larvicidal potency. However, statistical analysis indicated no significant differences between the treatments at any of the exposure times (*p* > 0.05), suggesting comparable overall efficacy under the experimental conditions.

### 3.3. Effect of *A. indica* Seed Oil Against *An. gambiae* s.l. Larvae in Semifield Conditions

The toxicity of *A. indica* seed oil against field-collected *Anopheles gambiae* s.l. larvae was evaluated in semifield conditions and compared with laboratory results. *A. indica* seed oil at the highest dose (75 ppm) resulted in 68.33% mortality in the target mosquito larvae after 72 h (Table 3).

### 3.4. Determination of LC_50_ and LC_90_ Values of Seed Oils Against *An. gambiae* s.l. Under Semifield Conditions

The dose–response relationship and the estimated LC_50_ and LC_90_ values, with 95% confidence limits, for *A. indica* seed oil at exposure times of 24, 48, and 72 h against early 4^th^-instar *An. gambiae* s.l. larvae are presented in Table 4. The toxicity effect of *A. indica* seed oil in the semifield trial was found to be less effective compared to the laboratory results. Specifically, the semifield data indicated that the larvae had higher LC_50_ and LC_90_ values for the EO than those observed in the laboratory experiment.

## 4. Discussion

There is a growing interest in exploring bioactive compounds from plants as potential alternatives to synthetic insecticides. Seed oils derived from plants contain various chemicals with biological activities, including larvicidal effects against mosquitoes. In the present study, the efficacy of *A. indica* and *S. molle* seed oils, along with their combinations, was evaluated against field-collected *Anopheles gambiae* s.l. early 4^th^-instar larvae under both laboratory and semifield conditions. The results showed that all tested seed oils were effective against the target larvae, with mortality rates being dose-dependent and influenced by exposure time with *A. indica* seed oil exhibiting the highest efficacy, followed by *S. molle* seed oils. The highest mortality was observed under laboratory conditions, with more than 50% mortality in all treatments at 75 ppm and 72-h exposure times. These findings emphasize the importance of concentration and exposure time for achieving effective mosquito control, and these findings also highlight that plant-derived seed oils are potential eco-friendly alternatives to synthetic larvicides. The high larvicidal activity of *A. indica* oil (LC_50_ = 1.04 ppm in the lab and 107 ppm in the semifield conditions) at 72 h showed promising results due to the presence of azadirachtin, a known insect growth regulator.

From the present study, we also observed that the combination treatment exhibited poor larvicidal efficacy. The lower efficacy of the combined *A. indica* and *S. molle* oils compared to individual oils may be due to possible antagonistic interactions between their active compounds, which could reduce overall toxicity. In addition, differences in chemical composition, concentration ratios, or potential competition at target sites in the larvae might contribute to this reduced effect. Further studies are recommended to investigate the interaction mechanisms of these oils in combination to optimize their use as botanical larvicides.

The observed larvicidal activity of *A. indica* EO was lower under semifield conditions compared to the laboratory results, and this is a commonly reported phenomenon in studies evaluating plant-derived larvicides. A similar trend was reported by a study conducted by [24], where higher mortality was observed in laboratory settings than in semifield trials using *Terminalia chebula* against various mosquito species, including *Aedes aegypti*, *Anopheles stephensi*, *Culex quinquefasciatus*, and *An. gambiae* s.s. These findings align with studies [17, 19, 25] which reported larvicidal and adulticidal effects of plant extracts, including *A. indica* and *S. molle*, against malaria vectors. Various factors, such as environmental conditions, oil volatility, and water quality, contribute to laboratory and field study result discrepancies.

The present findings demonstrate that *A. indica* seed oil exhibited stronger larvicidal activity against *An*. *gambiae s*.*l*. larvae than *S. molle* seed oil that might be attributed to the presence of azadirachtin, which is a known bioactive compound with potent insecticidal properties [26]. Similar larvicidal potential of *S. molle* has been documented in other studies. For example, researchers [27] reported that *S. molle* seed oil exhibited greater toxicity against *Culex pipiens* than other plant oils, while other researchers [28] documented a very noticeable insecticidal activity, with 96% mortality in *Haematobia irritans* larvae. Such variations among studies may be attributed to differences in oil composition influenced by geographic origin, extraction techniques, or the target mosquito species, all of which can affect the overall toxicity profile.

The seed oils demonstrated more effectiveness in laboratory studies compared to field tests. In the laboratory, *A. indica* showed superior larvicidal efficacy at all concentrations (25, 50, and 75 ppm) over the three exposure times. In semifield conditions, however, *A. indica* was less effective at all concentrations and exposure times, as indicated by the lower cumulative mortality after 3 days. This is one of the limitations of the present study which does not control the complexities of natural habitats, such as temperature fluctuations, wind, and water movement. This suggests that environmental factors in the semifield conditions may have influenced the efficacy of the EO, reducing its larvicidal activity compared to controlled laboratory conditions. Moreover, this could also be attributed to the increased resilience of field-collected mosquito larvae compared to laboratory-raised larvae [25, 29]. These results are consistent with previous studies [30], which highlighted the reduction in the longevity of *An. gambiae* s.l. adults using *A. indica* oil in field settings, which is a promising phenomenon in reducing malaria transmission. In conclusion, both *A. indica* and *S. molle* seed oils demonstrated significant larvicidal potential against *An. gambiae* s.l., with greater efficacy under laboratory conditions compared to semifield conditions. The differences in LC_50_and LC_90_values highlight the challenges of translating laboratory findings into field applications, emphasizing the need for formulation improvements and field validation for practical mosquito control programs.

## 5. Conclusion

The present study showed that, in laboratory settings, the seed oil from *A. indica* had the strongest larvicidal action against *An. gambiae* s.l. larvae, followed by *S. molle* and their combination. Although mortality rates were lower than in the lab, *A. indica's* effectiveness was still noteworthy in semifield settings. This is probably because environmental factors affect the stability and bioavailability of the oil. The findings imply that *A. indica* seed oil has potential as a botanical larvicide for integrated vector management systems, especially at higher doses. The optimization of concentrations for field application based on laboratory-derived LC_90_ values, evaluation of environmental persistence, and cost-effectiveness in comparison to traditional chemical larvicides should be the main objectives of future research [31].

## Figures and Tables

**Table 1:** Average ± SE of the percentage mortality of *An. gambiae s.l.* larvae in their 4^th^-instar after exposure to various concentrations of EOs and their mixtures.

Treatments	Concentration (ppm)	Average ± SE larval mortality at the time of exposure		
		24 h	48 h	72 h
*A. indica*	25	10 ± 0.00e	18.33 ± 0.33d	23.33 ± 0.33d
	50	26.66 ± 0.66c	48.33 ± 0.33b	56.66 ± 0.88b
	75	46.66 ± 0.33b	66.66 ± 0.88a	71.66 ± 0.88a

*S. molle*	25	6.66 ± 0.33e	16.65 ± 0.33c	16.66 ± 0.33e
	50	21.66 ± 0.33c	28.33 ± 0.66c	35 ± 0.57c
	75	33.33 ± 0.33b	41.66 ± 0.88b	51.66 ± 0.66b

*A. indica* and *S. molle*	25	8.33 ± 0.33e	10 ± 0.00e	18.33 ± 0.33e
	50	16.66 ± 0.88d	26.66 ± 0.88c	38.33 ± 0.66c
	75	26.66 ± 0.88c	48.33 ± 0.88b	55 ± 0.57b

Temephos/Abate	25	100.00 ± 0.00a	Nob	Nob
	50	100.00 ± 0.00a	Nob	Nob
	75	100 ± 0.00a	Nob	Nob

Control	Untreated	0.00 ± 0.00f	0.00 ± 0.00f	0.00 ± 0.00f

*Note:* Average within a column followed by different letters is significantly different; *p* < 0.05%; Tukey's honestly significant difference (HSD). Nob, no observation.

Abbreviation: SE, standard error.

**Table 2:** LC_50_ and LC_90_ values (with 95% confidence limits in parentheses) of seed oils under laboratory conditions after 24, 48, and 72 h of exposure against early 4th-instar field-collected *An. gambiae s.l.* larvae.

Plant oil	LC	Exposure hours		
		24 h	48 h	72 h
*A. indica*	50	1.60 (0.40–2.04)	1.07 (0.17–1.53)	1.04 (0.33–1.44)
	90	0.82 (0.02–1.33)	0.37 (0.00–0.801)	0.36 (0.02–0.72)
	*p* value	0.01	0.01	0.01

*S. molle*	50	1.75 (0.35–2.23)	1.54 (0.84–1.95)	1.17 (0.04–1.71)
	90	0.90 (0.00–1.44)	0.72 (0.12–1.12)	0.37 (0.00–0 0.85)
	*p* value	0.01	0.01	0.01

*A. indica* and *S. molle*	50	2.00 (0.20–2.66)	1.55 (0.91–1.95)	1.14 (0.10–1.65)
	90	1.02 (0.00–1.60)	0.76 (0.18–1.15)	0.37 (0.00–0.82)
	*p* value	0.03	0.03	0.03

Abbreviation: LC, lethal concentration.

**Table 3:** Average ± SE percentage mortality of field-collected *An. gambiae* s.l larvae due to different rates of *A. indica* seed oil and Temephos (Abate) with the chemical name of O,O′-(thiodi-4,1-phenylene) bis(O,O-dimethyl phosphorothioate) with three observation times in simulated field conditions.

Treatment	Concentration (ppm)	Average + SE at 24 h	Average + SE at 48 h	Average + SE at 72 h
*A. indica* seed oil	25	8.33 ± 0.33d	16.66 ± 0.33c	21.66 ± 0.33c
	50	23.33 ± 0.33c	46.00 ± 0.57b	53.33 ± 0.33b
	75	43.33 ± 0.33b	63.33 ± 0,33a	68.33 ± 0.66a

Temephos (Abate)	25	100 ± 0.00a	Nob	Nob
	50	100 ± 0.00a	Nob	Nob
	75	100 ± 0.00a	Nob	Nob

Control	Untreated	0.00 ± 0.00e	0.00 ± 0.00d	00 ± 0.00d

*Note:* Average within a column followed by a different letter is significantly different *p* < 0.05%, Tukey's honestly significant difference (HSD) test. Nob = no observation.

**Table 4:** LC_50_ and LC_90_ values (with 95% confidence limits in parentheses) of *A. indica* seed oil under semifield conditions after 24, 48, and 72 h of exposure against early 4^th^-instar field-collected *An. gambiae* s.l. larvae.

Treatment	Exposure hours					
	24 h		48 h		72 h	
*A. indica* seed oil	LC_50_	LC_90_	LC_50_	LC_90_	LC_50_	LC_90_
	1.66 (0.40–2.12)	0.858 (0.01–1.38)	1.11 (0.11–1.59)	0.38 (0.00–0.83)	1.07 (0.30–1.48)	0.36 (0.01–0.74)

Abbreviation: LC, lethal concentration.

## Data Availability

The datasets used and/or analyzed during the current study are available from the corresponding author on reasonable request.
